# Effects of long-term cocaine self-administration on brain resting-state functional connectivity in nonhuman primates

**DOI:** 10.1038/s41398-020-01101-z

**Published:** 2020-12-02

**Authors:** Stephen J. Kohut, Dionyssios Mintzopoulos, Brian D. Kangas, Hannah Shields, Kelly Brown, Timothy E. Gillis, Michael L. Rohan, Jack Bergman, Marc J. Kaufman

**Affiliations:** 1 Behavioral Biology Program, McLean Hospital, Belmont, MA 02478 USA; 2 McLean Imaging Center, McLean Hospital, Belmont, MA 02478 USA; 3 Department of Psychiatry, Harvard Medical School, Boston, MA 02115 USA; 4 Translational Imaging Laboratory, McLean Hospital, Belmont, MA 02478 USA

**Keywords:** Molecular neuroscience, Psychology

## Abstract

Long-term cocaine use is associated with a variety of neural and behavioral deficits that impact daily function. This study was conducted to examine the effects of chronic cocaine self-administration on resting-state functional connectivity of the dorsal anterior cingulate (dACC) and putamen—two brain regions involved in cognitive function and motoric behavior—identified in a whole brain analysis. Six adult male squirrel monkeys self-administered cocaine (0.32 mg/kg/inj) over 140 sessions. Six additional monkeys that had not received any drug treatment for ~1.5 years served as drug-free controls. Resting-state fMRI imaging sessions at 9.4 Tesla were conducted under isoflurane anesthesia. Functional connectivity maps were derived using seed regions placed in the left dACC or putamen. Results show that cocaine maintained robust self-administration with an average total intake of 367 mg/kg (range: 299–424 mg/kg). In the cocaine group, functional connectivity between the dACC seed and regions primarily involved in motoric behavior was weaker, whereas connectivity between the dACC seed and areas implicated in reward and cognitive processing was stronger. In the putamen seed, weaker widespread connectivity was found between the putamen and other motor regions as well as with prefrontal areas that regulate higher-order executive function; stronger connectivity was found with reward-related regions. dACC connectivity was associated with total cocaine intake. These data indicate that functional connectivity between regions involved in motor, reward, and cognitive processing differed between subjects with recent histories of cocaine self-administration and controls; in dACC, connectivity appears to be related to cumulative cocaine dosage during chronic exposure.

## Introduction

Cocaine use disorder is a significant public health problem that is associated with medical complications and societal issues of unemployment and high crime rates. The 2017 National Survey on Drug Use and Health reported that cocaine continues to rank among the most-heavily used illicit drugs, and the number of cocaine dependent individuals has remained constant over the last decade^1^. In addition to its societal impact, long-term cocaine intake has been associated with neuroadaptations that may play a role in a variety of behavioral deficits that impact daily functioning of users (reviewed by Potvin et al.^2^). Further characterizations of such neuroadaptations may lead to improved treatment strategies to mitigate the deleterious effects of long-term cocaine use.

The acute reinforcing or subjective effects of cocaine have been attributed to its pharmacological action at dopaminergic sites in striatal regions including the nucleus accumbens and basal ganglia^3^. However, repeated drug use induces neuroadaptations in those neural circuits^4^, which may contribute to the expression of a number of addiction-like behaviors, including deficits in cognitive-behavioral control and persistent or uncontrolled drug use^5^. Functional neuroimaging studies have generally supported the idea that changes in subcortical structures involved in reward processing and cortical areas involved in cognitive-behavioral control converge to maintain persistent drug-taking behavior^6–8^. Moreover, neurocircuit-based models of addiction have focused on mesocorticolimbic pathway dysfunction^6,9,10^ as a key mediator of the addiction process. For example, glutamatergic projections between cortical and subcortical regions have been shown to strengthen the neural response to drug-related stimuli^7,11^.

Two brain regions that play important roles in behavioral control and reward-related learning and decision-making are the dorsal anterior cingulate cortex (dACC) and putamen. The dACC serves as a hub of the salience network which integrates information about the environment to guide attention^12^. Neuroimaging studies have found that dACC reactivity is altered in response to drug-associated stimuli or during task performance in substance users, suggesting the importance of this network in drug addiction (reviewed by Goldstein and Volkow^13^). For example, Maas et al.^14^ have noted that dACC activity increases in the presence of cocaine-related stimuli in crack-cocaine users. Other investigators have reported that, compared to control subjects, cocaine users display ACC hypoactivity during a go/go-no task^15,16^ and, furthermore, that the strength of dACC-nucleus accumbens connectivity can be related to cognitive control (see also ref. ^17^). Investigations of resting-state functional connectivity, in which the brain’s functional organization is inferred by measuring the correlation of spontaneous fluctuations of signals between various brain regions^18,19^, have supported a relationship between cocaine exposure and altered resting dACC functional connectivity. Motzkin et al.^20^, for example, documented differences in dACC-nucleus accumbens connectivity in prison inmates diagnosed with substance use disorder (see also refs. ^8,21^).

The putamen, a brain region involved in motor control and reinforcement learning^22^, is a target for the pharmacological actions of cocaine due to its high density of dopamine transporters. Thus, acute administration of cocaine to humans or nonhuman primates^23,24^ induces functional inhibition of putamen and other striatal or motor regions (but see refs. ^25,26^). Long-term cocaine use has been shown to produce long-lasting neuroadaptations in the putamen. For example, using magnetic resonance spectroscopy in nonhuman primates exposed to noncontingent cocaine injections for 9-months, Liu et al.^27^ found that glutamate/total creatine (tCr) and glutamine/tCr metabolite ratios in putamen increased in a time-dependent manner. Positron emission tomography (PET) studies with dopaminergic radiotracers also have reported decreased DA D_2_ receptor density in the putamen of chronic cocaine users^28,29^. These neuroadaptations are consistent with reports of drug craving being positively correlated with neural activity in the putamen^30^.

The present study was conducted to examine the relationship between long-term cocaine self-administration and dACC or putamen whole brain functional connectivity (rsFC). Previous studies have primarily focused on activity of these regions during task-related events or strength of functional connectivity to specific brain regions within the mesocorticolimbic circuit. In the present study, a seed-region analysis was used to gain insight into functional connectivity with these two regions after long-term cocaine self-administration. To accomplish this, resting-state fMRI was acquired in squirrel monkeys following 140 sessions of high dose cocaine self-administration and compared to fMRI data from control subjects. Scans were acquired at ultra-high magnetic field (9.4 Tesla), which provides higher spatial resolution of functional circuits under treatment conditions^31^.

## Materials and methods

### Subjects

Two groups of six adult male squirrel monkeys (*Saimiri sciureus*) served as subjects in this study. Five of the subjects in the cocaine self-administration group previously participated in studies of the neurochemical effects of passive cocaine administration^27^ but had not received cocaine or other drug treatment for ~2 years prior to the present studies; the sixth subject was drug and experimentally naive. A report of the effects of chronic cocaine self-administration and N-acetylcysteine on learning, cognitive flexibility, and reinstatement in this group of subjects has been recently published (see ref. ^32^). Six additional drug-experienced monkeys that had not received drug treatment for ~1.5 years, served as controls. This sample size is consistent with previously published literature showing effects of drug exposure on neuroimaging endpoints^24,26,27,32,33^. Subjects were not randomized into group and there was no blinding to experimental conditions.

Subjects were pair-housed in a temperature- and humidity-controlled vivarium with a 12-h light/dark cycle (0700–1900). Monkeys had unlimited access to water in the home cage and were maintained at approximate free-feeding weights with a nutritionally balanced diet of high protein biscuits (Purina Monkey Chow, St. Louis, MO). Fresh fruit and nuts were provided as part of a daily environmental enrichment plan. Experimental sessions were conducted 5 days a week. The protocol was approved by the Institutional Animal Care and Use Committee at McLean Hospital in a facility licensed by the US Department of Agriculture and in accordance with guidelines provided by the Committee on Care and Use of Laboratory Animals of the Institute of Laboratory Animals Resources, Commission on Life Sciences^34^.

### Cocaine self-administration

The self-administration protocol used for the present study was described in detail previously^32^. Briefly, subjects sat in a Plexiglas chair housed within a ventilated, sound-attenuating enclosure during daily self-administration sessions. A panel containing two response levers, colored stimulus lights, and a Plexiglas receptacle (5 × 3.5 × 1.27 cm) was mounted in front of the seated subject. Each press of one lever produced an audible click and was recorded as a response. An infusion pump (PHM-100-10, Med Associates, St. Albans, VT), located outside the enclosure, was used to deliver intravenous cocaine injections. Subjects responded on the lever under a 10-response fixed ratio (FR10); timeout (TO) 60 s schedule of IV cocaine self-administration. The unit dose that resulted in maximum average daily intake (0.32 mg/kg/inj) determined during the evaluation of the cocaine dose-effect function (see ref. ^32^) was then available during daily self-administration sessions conducted Monday–Friday for 140 sessions. Daily intake was limited to 3.2 mg/kg cocaine to minimize the potential for adverse effects. All experimental events and data collection were controlled by Med Associates software (MedPC v4.2).

### Magnetic resonance imaging (MRI)

Magnetic resonance imaging (MRI) scans were acquired using a 9.4 Tesla horizontal bore magnet system (Varian Direct Drive, Varian Inc, Palo Alto, CA, USA) running Vnmrj software (version 3.2A). A 12 cm inner diameter gradient was used with maximum gradient strength of 40 G/cm; a custom-made quadrature volume coil that was designed for squirrel monkey brain scans for optimal magnetic field homogeneity was used. Each coil channel was tuned and matched separately.

To minimize the influence of acute effects of cocaine on brain activity, scan sessions were conducted 48–72 h after the last self-administration session (i.e., session 140). Monkeys were transported from the vivarium to an animal preparation room within the scanner suite. After ~30-min of acclimation, subjects were sedated with 10 mg/kg ketamine, IM for anesthesia induction, intubated, and then maintained on 1–1.2% isoflurane gas throughout the scanning procedures. A circulating warm-water blanket and fleece wrap were used to maintain body temperature. Monkeys were scanned in the prone position in an MR-compatible monkey holder. Vital signs such as heart rate, respiration rate, body temperature, and oxygen saturation (SpO_2_) were monitored and maintained throughout the procedure by trained technical staff.

Monkeys were scanned with the following pulse sequences: *Structural MRI scan*: 2D fast spin echo scan in matching slices and orientation with the fMRI acquisition. Repetition (TR) and echo times (TE) were optimized to provide good tissue contrast (TR/TE = 6.5 s/11 ms, in-plane acquisition matrix 128 × 128, 64-mm field of view (FOV), fast spin echo factor 8, 3 averages, 34 slices, 1-mm slice thickness, voxel size = 0.5 × 0.5 × 1 mm. Time 5:25 min). *fMRI scan (resting-state fMRI):* Gradient-echo EPI (TR/TE = 2 s/17 ms, in-plane acquisition matrix 64 × 64, 64 mm in-plane FOV, 34 slices, 1-mm slice thickness, voxel size = 1  × 1  × 1 mm, 416.7 kHz EPI bandwidth, 0.216 ms EPI echo spacing, 360 volumes in the fMRI timeseries. Time: 12:00 min). The fMRI sequence was run for 5 min without data prior to the acquisition to equilibrate gradient heating and minimize artifact drift. *Anatomic T1/fieldmap MRI scan:* Three three-dimensional gradient-echo FLASH images (TR = 30 ms, TE = 1.50 ms, 1.88 ms, 3.20 ms, 64 × 64 × 64 matrix on 64 mm FOV oriented as the fMRI images. Time 2:03 min × 3).

### MRI data processing

Standard image preprocessing was performed within the FSL software library (FMRIB, Oxford University, UK^35^). During preprocessing, the fMRI timeseries images were first rotated to be in the same orientation as the reference T1w image from the VALiDATe squirrel monkey template^36^ and then motion-corrected. A 150 s temporal filter, spatial smoothing using a Gaussian kernel of 2.0 mm FWHM, and global 4D mean intensity normalization were used during preprocessing. For registration to standard space, whole brain masks were created for individual subjects for skull-stripping followed by manual editing and visual inspection. The extracted brain was then aligned to the VALiDATe T1w template through a 12 DOF affine transformation. Region of interest seed areas (seed ROIs), identified from ref. ^37^, were drawn on the reference image at the level of the left dACC and putamen. A white matter (WM) mask was drawn from the VALiDATe FA squirrel monkey template (see ref. ^36^).

### Data analysis

For cocaine self-administration data, the primary dependent measure was total session cocaine intake (mg/kg) calculated by multiplying the total number of infusions/session by the unit dose (mg/kg/inj) of self-administered cocaine.

For region of interest analyses^38^, mean timeseries for the dACC and putamen ROIs were extracted for each subject and separate whole brain fMRI connectivity analyses was performed using the seed mean timeseries and the nuisance WM timeseries and 6 motion correction positional results as covariates (FSL Feat). Individual connectivity maps were derived using a Z threshold = 3.1. An image-based group comparison was performed between the control squirrel monkey fMRI and chronic cocaine self-administration squirrel monkey fMRI images using an unpaired *t*-test (FSL feat).

To explore whether different resting-state connectivity patterns with the dACC or putamen seed regions were associated with total drug intake over 140 sessions, mean regional connectivity for each individual subject analysis were extracted from the seed ROIs, compared with the control group mean as a percent difference, and plotted as a function of total cocaine intake (mg/kg). Correlational analyses were then accomplished using standard regression (GraphPad Prism version 8, San Diego, CA) to determine the relationship between seed connectivity and drug intake. Outliers were identified using the ROUT method in GraphPad Prism. This method is based on the false discovery rate (FDR) and the chance of falsely identifying one or more outliers was set at 4%^39^. All data met the assumptions of the statistical tests utilized and individual behavioral and neuroimaging data are shown in Fig. 4.

### Drugs

Cocaine hydrochloride was purchased from Sigma Pharmaceuticals (St. Louis, MO) and prepared in 0.9% saline solution. Drug concentrations for self-administration experiments were filtered and prepared for each subject so as to deliver the unit dose of IV cocaine in a 0.1 ml infusion. Cocaine doses are expressed in terms of the free base weight.

## Results

**Fig. 1:**
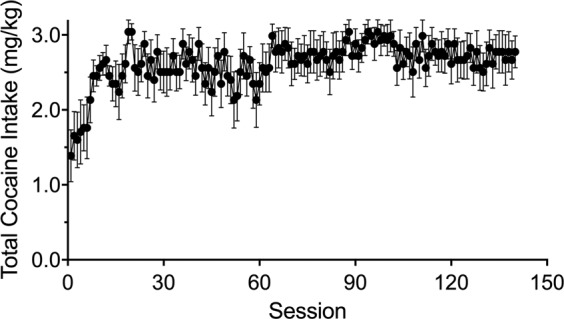
Average daily cocaine intake (mg/kg) over 140 sessions of 0.32 mg/kg/inj cocaine self-administration in squirrel monkeys. Data shown are the mean +/− SEM of the group (*n* = 6).

Cocaine maintained robust self-administration in all subjects over the course of 140 sessions. Figure 1 shows that 0.32 mg/kg/inj cocaine engendered ~1.5 mg/kg/session intake during the first several sessions which gradually increased over a 2-week period to ~2.7 mg/kg/session. Daily intake was then relatively stable throughout the 140-session period. The average total intake for the group over the 140 sessions of self-administration was 367 (+/−19.9) mg/kg.

**Fig. 2:**
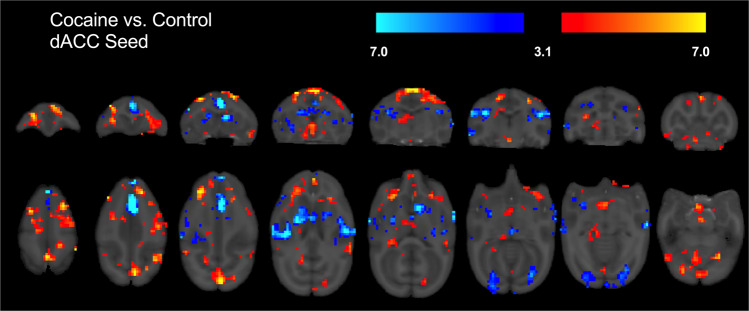
Significant differences in functional connectivity between the cocaine self-administration group and control subjects in dACC seed region. Cocaine > control is shown in red/yellow while cocaine < control is shown in blue. Additional details are shown in Table 1.

**Fig. 3:**
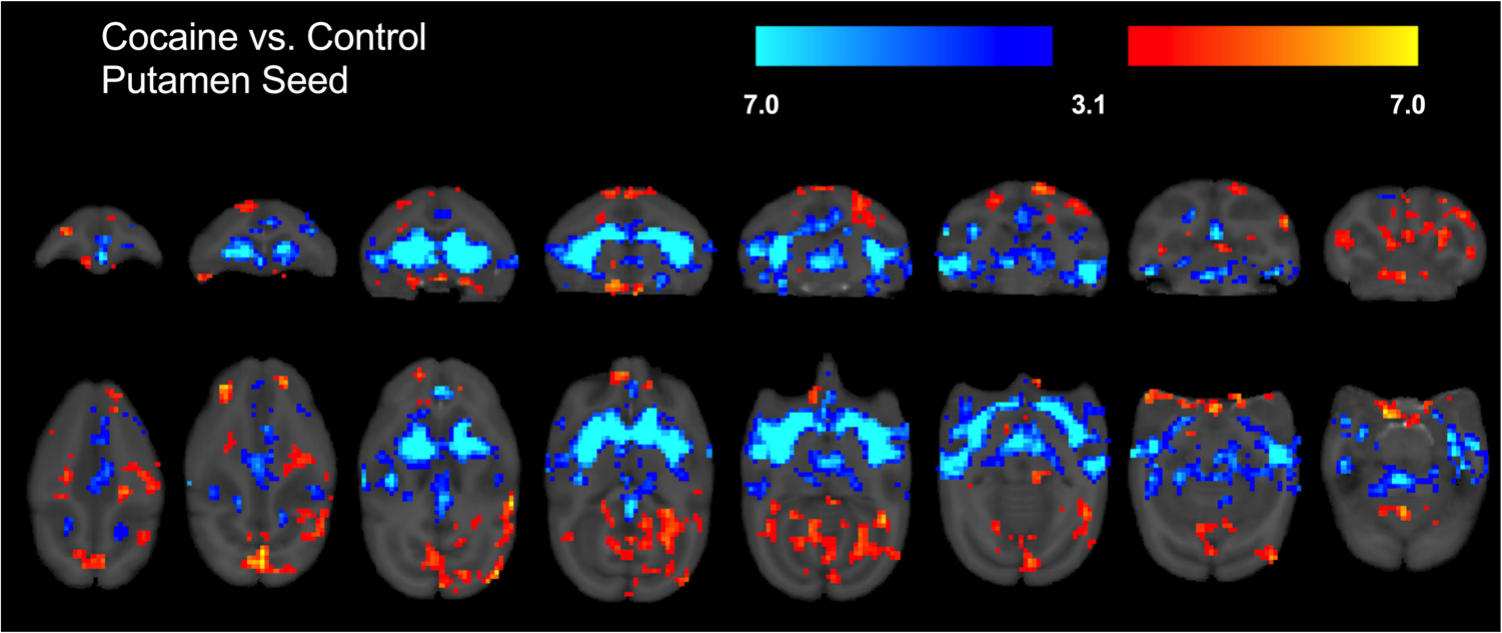
Significant differences in functional connectivity between the cocaine self-administration group and control subjects in putamen seed region. Cocaine > control is shown in red/yellow while cocaine < control is shown in blue. Additional details are shown in Table 2.

Whole brain group (cocaine vs control) differences in functional connectivity between the two seed ROIs and whole brain are shown in Figs. 2 and 3 and detailed in Tables 1 and 2. In the cocaine group, weaker functional connectivity was found between the dACC seed and local ACC regions. Weaker functional connectivity also was found between the dACC seed and regions primarily involved in motor behavior such as the left caudate, putamen, and ventral premotor areas. Conversely, stronger connectivity of the dACC in the cocaine group was found with areas implicated in reward (i.e., nucleus accumbens, medial and lateral septum, and orbito-frontal area) and with cognitive processing (e.g., medial, dorso-medial, and dorso-lateral prefrontal areas); *p*’s < 0.05. For the putamen seed, the cocaine group exhibited weaker widespread connectivity with subcortical regions including caudate, ventral premotor, insula, globus pallidus, and with various subregions of the anterior cingulate. Stronger putamen seed connectivity was found with areas implicated in reward (i.e., nucleus accumbens, several medial orbito-frontal regions, temporal/parietal cortices) and motor behavior (dorsal premotor, primary, and supplementary motor cortices); all *p*’s < 0.05.

**Table 1:** Summary of group differences in resting-state functional connectivity between cocaine self-administering and control squirrel monkeys from dACC seed.

			VALiDATe29 atlas coordinates				
Cluster	# Voxels	*P*	Z-MAX	*X* (mm)	*Y* (mm)	*Z* (mm)	Brain regions
*Cocaine* *>* *control*							
20	549	6.33E−24	11.4	−1.69	7.26	14.6	Supplementary Motor Area, dorsal premotor, primary motor cortex, medial parietal area
19	339	3.27E−17	10.1	−0.694	−21.6	7.11	Visual areas
18	322	1.29E−16	6.01	−5.66	−14.6	−5.05	Visual areas
17	224	6.16E−13	5.68	1.29	6.26	−6.92	Nucleus accumbens, medial, lateral, ventral septum
16	173	8.23E−11	7.25	6.26	18.2	7.11	(R) prefrontal areas (lateral PFC, dorsal PFC, ventral PFC, lateral/medial OFC)
15	90	8.34E−07	5.32	−8.64	16.2	2.44	(L) dorsal/ventral PFC
14	58	5.85E−05	6.74	4.27	−10.6	6.18	(R) medial superior temporal area
13	56	7.82E−05	4.67	2.29	−4.67	−0.371	Cerebellum/visual areas
12	47	0.000303	7.18	−4.67	17.2	9.92	(L) lateral, dorsal PFC
11	35	0.00212	4.52	14.2	−14.6	−6.92	(R) visual area
10	31	0.00426	4.53	4.27	23.2	6.18	(R) dorsal/ventral PFC
9	29	0.0061	5.09	8.25	−2.68	2.44	(R) medial superior temporal area
8	28	0.00732	5.08	−0.694	1.29	−6.92	(B) visual areas
7	24	0.0155	4.59	−4.67	13.2	−4.11	(L) nucleus accumbens
6	23	0.0188	5.84	−9.64	−6.66	−9.72	Brainstem
5	22	0.0229	6.21	−0.694	23.2	5.24	(R) mOFC
4	21	0.028	5.21	−5.66	−19.6	4.31	(L) visual areas
3	21	0.028	6.62	−3.68	14.2	6.18	(L) dorso-lateral prefrontal area, arcuate sulcus
2	21	0.028	4.77	−14.6	11.2	−3.18	(L) ventral premotor area (?)
1	19	0.0419	5.05	13.2	−7.65	8.05	(R) temporo-parietal area
*Cocaine* *<* *control*							
10	691	5.67E−28	15.5	−1.69	11.2	8.98	Cingulate
9	161	2.78E−10	7.74	−13.6	1.29	4.31	(L) Somatosensory
8	128	9.40E−09	5.43	7.26	−23.5	0.565	(R) visual area
7	115	5.96E−08	6.66	−8.64	−19.6	−0.371	(L) visual area
6	43	0.000568	6.62	17.2	2.29	−3.18	(R) Area 44 - IFG
5	33	0.003	7.61	−17.6	−0.694	−1.31	(L) ventral premotor
4	30	0.0051	8.6	−1.69	22.2	9.92	mPFC
3	29	0.0061	7.86	−17.6	7.26	3.37	(L) superior temporal sulcus
2	24	0.0155	5.38	0.3	−0.694	1.5	PAG
1	23	0.0188	7.05	13.2	11.2	7.11	(R) Dorsal premotor

Regions identified from refs. ^36,37^.

*R* right, *L* left, *B* bilateral.

**Table 2:** Summary of group differences in resting-state functional connectivity between cocaine self-administering and control squirrel monkeys from putamen seed.

			VALiDATe29 atlas coordinates				
Cluster	# Voxels	*P*	Z-MAX	*X* (mm)	*Y* (mm)	*Z* (mm)	Brain regions
*Cocaine* *>* *control*							
9	1257	1.40E−38	7.05	−0.694	−18.6	8.05	(B) visual areas
8	553	3.96E−22	6.45	−5.66	2.29	14.6	(L) primary motor cortex, superior parietal, dorsal prelunate gyrus
7	167	1.02E−09	7.68	−4.67	9.24	−7.85	(L) nucleus accumbens
6	90	2.74E−06	7.05	6.26	20.2	7.11	(R) dorsal-lateral PFC
5	54	0.000242	4.86	11.2	−6.66	−0.371	(R) middle temporal area, visual areas
4	50	0.00042	6.45	−5.66	20.2	8.98	(L) dorsal PFC, mOFC
3	30	0.00859	5.58	−12.6	−21.6	−4.11	(L) visual area
2	28	0.012	4.88	−1.69	−5.66	0.565	(B) posterior cingulate
1	25	0.02	5.47	14.2	13.2	−4.11	(R) orbito-frontal cortex
*Cocaine* *<* *control*							
6	4449	0	21.4	6.26	11.2	1.5	(B) putamen, caudate, insula, ventral premotor, anterior cingulate, globus pallidus, paraventricular hypothalamic nucleus, posterior cingulate, superior temporal lobe, hippocampal nuclei (CA1)/dentate gyrus, medial prefrontal area
5	84	5.54E−06	7.91	5.27	−10.6	12.7	(R) lateral, ventral intraparietal area
4	31	0.00729	5.75	−10.6	14.2	7.11	(L) dorsal, ventral PFC
3	26	0.0169	4.75	−0.694	−16.6	−10.7	Brainstem
2	26	0.0169	5.52	−4.67	−9.64	8.98	(L) lateral, ventral intraparietal area
1	24	0.0239	5.11	−8.64	−18.6	−9.72	Brainstem

Regions identified from refs. ^36,37^.

*R* right, *L* left, *B* bilateral.

**Fig. 4:**
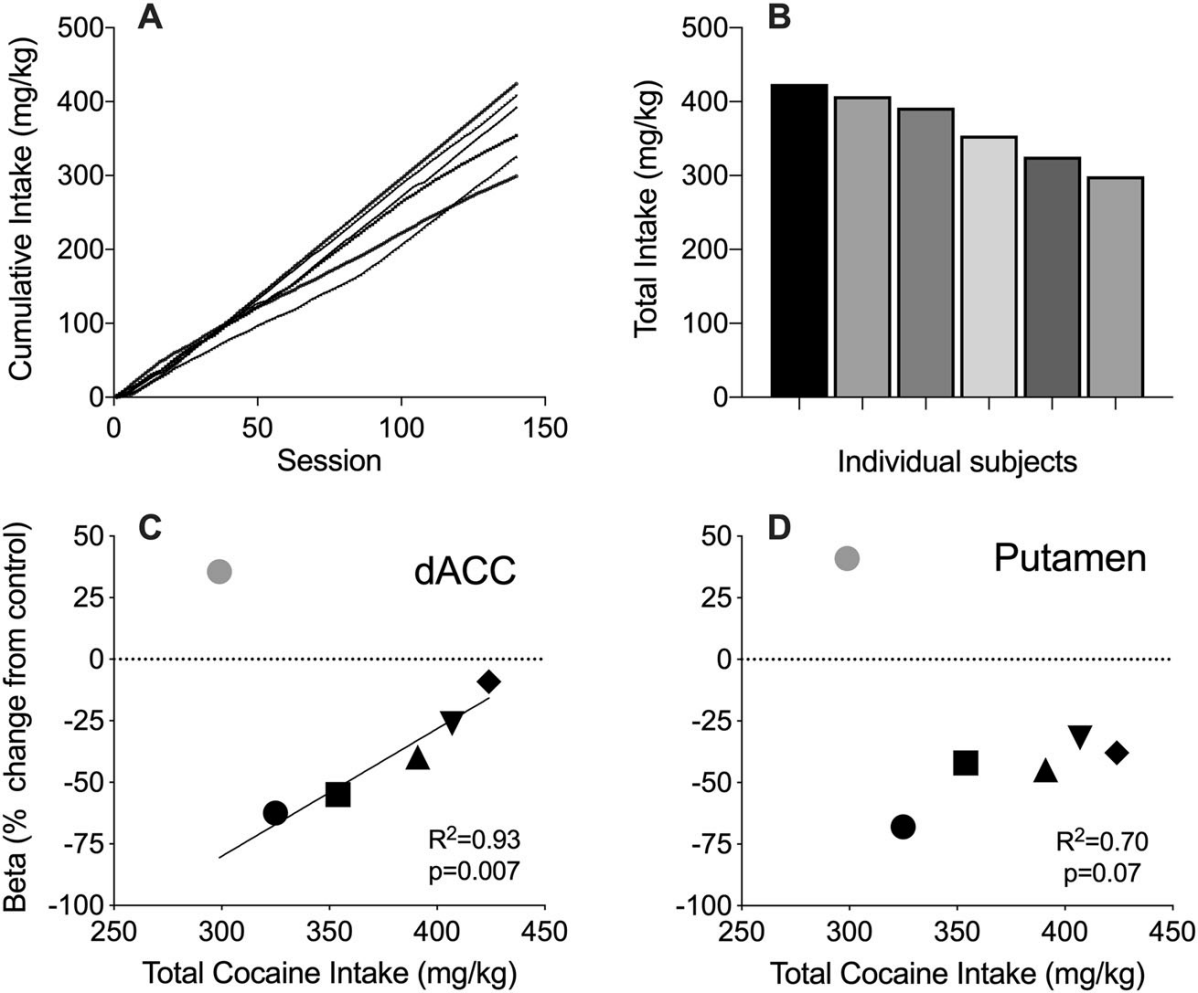
Relationship between cocaine intake and functional connectivity with dACC or putamen. Top panels: Individual differences in cumulative (**A**) and total (**B**) cocaine intake (mg/kg) over 140 sessions of 0.32mg/kg/inj cocaine self-administration in squirrel monkeys. Each line (left panel) or bar (right panel) represents individual subject data. Bottom panels: Percent difference from control group in connectivity with dACC (**C**) and putamen (**D**) for individual subjects in the cocaine self-administration group plotted as a function of total cocaine intake (mg/kg). Each data point represents the relationship for an individual subject. The gray symbol indicates data points identified as outliers by the regression analyses. The solid line represents a standard regression fit to non-outlier data.

Figure 4A shows cumulative cocaine intake for individual subjects across daily sessions. Cocaine self-administration was largely invariant across the first 50 sessions, with each subject earning ~100 mg/kg over that period. Interestingly, after session 50, differences in rate of cumulative intake across subjects became evident such that total cumulative cocaine intake varied among subjects by 125 mg/kg. Figure 4B shows the range (299–424 mg/kg) of total cocaine intake among individual subjects over the 140 sessions. To explore whether total drug intake was associated with differences in dACC or putamen seed region resting-state connectivity, connectivity beta weights from each individual were extracted from the seed ROIs and plotted as a function of total cocaine intake (mg/kg). The regression removed one subject that was identified as an outlier (gray symbol) in both analyses. Results show a significant association between total cocaine intake and strength of connectivity with the dACC (*R*2 = 0.93; *p* = 0.007; Fig. 4C), but not putamen (*R*2 = 0.70; *p* = 0.07; Fig. 4D). Interestingly, this was an inverse association: strength of connectivity with the dACC in subjects that self-administered the highest levels of cocaine was more like the control group whereas subjects that had low to intermediate cocaine intake displayed the lowest connectivity, relative to controls.

## Discussion

The present study was designed to assess differences in resting-state functional connectivity associated with long-term cocaine self-administration in adult male squirrel monkeys. Overall, the present results indicate a selective pattern of resting-state functional connectivity differences with seed regions that encompass a number of brain circuits previously demonstrated to be altered in human cocaine users. Further, connectivity strength of dACC, but not putamen, was related to total cocaine intake, suggesting that the dACC may be a key hub mediating abnormal patterns of brain activity and behavior following long-term cocaine use.

The cocaine self-administration protocol used in this study involved a relatively high unit dose of cocaine (0.32 mg/kg/inj), positioned on the descending limb of the cocaine dose-response function (see ref. ^32^), that maintained robust, high levels of intake over the 140 sessions. Interestingly, although daily intake was ~1.5 mg/kg/session for several sessions following the initial acquisition of cocaine self-administration, intake gradually increased by almost 50% (~2.7 mg/kg/session) over the first two weeks of the chronic cocaine self-administration protocol. The increase in total intake suggests that exposure to the high dose of cocaine produced neuroadaptations or tolerance to the direct, rate-limiting effects of cocaine^40^. In fact, following gradual increases in cocaine intake for ~2 weeks, stability in daily intake was achieved after ~60 sessions, albeit at a higher intake level. These findings are similar to reports of escalated drug-taking in human users^41^, which supports the idea that the protocol used here is translationally relevant for studying neural adaptions in response to chronic cocaine exposure.

Previous studies in human substance users, including cocaine users, have consistently demonstrated dACC hypofunctionality, both during task performance and in evaluations of resting-state connectivity^15,16,20,42,43^. For example, weaker connectivity among several subregions of the ACC, including dACC, has been reported in heroin-dependent individuals^42^. Further, reduced rsFC between dACC and other components of the salience network^44^ as well as between the cingulate and striatum^8^ has been reported in non-treatment seeking cocaine users. The present results are consistent with those findings in that local dACC seed connectivity with adjacent structures such as caudate and somatosensory cortex was weaker following extended cocaine self-administration. In contrast, stronger connectivity was found between the dACC and several other regions of the prefrontal cortex including orbital, medial, ventro-lateral, dorso-lateral, and dorso-medial prefrontal areas. The stronger connectivity with other regions involved in higher-order cognitive processing appears to be related to long-term cocaine self-administration and may represent the consequences of decreased local ACC connectivity and the impairment of associated processes relevant to behavioral inhibition^8,15^. In line with this idea, we recently reported that, during long-term cocaine self-administration in the present subjects, learning and cognitive flexibility were disrupted for ~30 sessions but eventually regained pre-exposure levels of performance^32^. Perhaps, as discussed above, the recruitment of activity from prefrontal executive structures helped to overcome initial deficits in dACC function to eventually improve task performance. In this regard, studies in human subjects have demonstrated similar recruitment in tasks requiring behavioral adaptation and attentional control^45,46^. Additional studies to document the development of changes in dACC functional connectivity or task-related fMRI designs are needed to address this question.

The pattern of putamen connectivity in monkeys self-administering cocaine—i.e., weaker functional connectivity between the putamen and subcortical regions (e.g., caudate, insula, hippocampus, globus pallidus, septum, paraventricular hypothalamus) as well as ventral premotor cortex—is similar to the pattern of functional inhibition of putamen and other subcortical regions observed after acute exposure to cocaine in humans^47,48^ and nonhuman primates^24^. Inasmuch as cocaine’s elimination half-life is ~1.5–4 h^49^ and fMRI data in the present study were obtained at least 48 h after the last cocaine self-administration session, it is unlikely that the effects described here were due to residual cocaine or metabolites in the system but rather, reflect neuroadaptations that persist after cocaine use. Such neuroadaptations may be related to other types of changes that have been reported to follow long-term cocaine exposure, e.g., abnormalities in glutamate regulation that may reflect alterations in synaptic plasticity^27,50–52^ or reductions in dopamine D2 receptor availability in the putamen of abstinent cocaine users^29,33^ that may underlie a heightened propensity for relapse even after longstanding abstinence^48,53^. rsFC evaluations further support this view, as strength of connectivity between putamen and insula has been shown to predict relapse rate in outpatient substance users^54^.

A hallmark feature of cocaine use disorder is the loss of voluntary control over drug-seeking and drug-taking behavior that drives persistent drug use^55^. Evidence suggests that dysregulation of drug-seeking and drug-taking is the result of altered connections between limbic and cortical structures. The present study identified stronger connectivity between putamen and several cortical regions that are distinct from those found with the dACC seed and appear to be centered around the orbital prefrontal cortex (OFC; see Table 1), an area often reported as altered in cocaine users (see refs. ^56–58^; reviewed by London et al.^59^ and Olausson et al.^60^). The OFC is thought to play a primary role in monitoring the environment and modulating behavior based on changing reinforcement contingencies^61^. Of importance to the present results, the OFC has direct connections with several subcortical regions involved in reward processing. These include the nucleus accumbens, a region in which functional connectivity with putamen was stronger in animals that had self-administered cocaine. Taken together, these findings are consistent with the idea that functional circuits between cortical and limbic regions can become strengthened as a consequence of long-term cocaine use.

As mentioned, the present data were collected in a multimodal study to examine the effects of long-term daily cocaine self-administration on both behavioral and neuroimaging endpoints. We previously reported that total cocaine intake was directly associated with impaired performance in a touchscreen-based model of repeated acquisition (i.e., learning) following 30 days of cocaine self-administration^32^. We sought to extend that finding by determining whether the strength of connectivity with the seed regions studied here also might be directly related to the level of cocaine intake. Previous studies have demonstrated that rsFC between dACC and striatal regions correlated negatively with severity of nicotine addiction^62,63^ and gray matter volume in dACC has been shown to be related to severity of cocaine dependence^64^. Surprisingly, our results show that cocaine intake was inversely related to the strength of dACC connectivity; no relationship between cocaine intake and putamen connectivity was observed. These observations are intriguing and suggest that the relationship between intake and dACC connectivity also may be related to stimulus-reward learning associated with drug self-administration experience. Of note, the subject with the lowest total cocaine intake (mg/kg) showed the strongest dACC connectivity which may suggest that a threshold of cocaine history is necessary before the inverse relationship between cocaine intake and dACC connectivity is evident. However, these suggestions are speculative, as the strength of dACC connectivity was not determined prior to cocaine self-administration. Thus, although all subjects readily acquired cocaine self-administration, which remained stable throughout the 140-session experimental period, it is unknown whether there is a relationship between baseline strength of connectivity and the speed of acquisition, or the amount of cocaine self-administered. Finally, as discussed above, it is possible that increased connectivity with other prefrontal regions helped normalize local connectivity with the dACC. Additional studies tracking changes in both local and regional circuits during the course of self-administration may provide a more global understanding of the neural impact of cocaine exposure.

While the present study offers important new insights into local and regional neural activity in subjects with a history of long-term cocaine self-administration, some caveats deserve mention. First, time-course experiments were outside the scope of the present study and, consequently, only one timepoint was assessed. Thus, time courses and durability of changes in rsFC are unknown^65^ and ought to be assessed in future research. Second, while the strong correlation between cocaine intake and dACC connectivity suggests that these results likely represent the effects of long-term cocaine self-administration, the control group used here was not engaged in behavioral studies during the 140-session period, and the influence of operant learning alone on dACC connectivity is unknown. Future studies of behavior maintained by other reinforcers, e.g., food delivery, are needed to investigate this question. Third, the present study utilized only male subjects, and the degree to which similar results might be obtained in female subjects is unclear. With growing recognition of sex-related differences in the behavioral and neural responses to drugs^66,67^, including cocaine^68–75^, this also will be an important area for further investigation. Finally, rsFC in the present study was determined in subjects that were lightly anesthetized with isoflurane (1–1.2%). Previous evaluations of the influence of isoflurane on intrinsic connectivity in nonhuman primates showed that patterns of rsFC are stable at low levels of isoflurane (1–1.5%^76^). Thus, it is likely that isoflurane anesthesia in the present study did not affect rsFC in a meaningful way (see also ref. ^77^). In view of similarities in the functional organization of brain networks across primate species^31,78^, the present findings may provide a unique translational perspective for understanding the neural impact of extended cocaine use in humans.
